# Stat3-positive tumor cells contribute to vessels neoformation in primary central nervous system lymphoma

**DOI:** 10.18632/oncotarget.16115

**Published:** 2017-03-10

**Authors:** Simona Ruggieri, Roberto Tamma, Nicoletta Resta, Francesco Albano, Nicoletta Coccaro, Daria Loconte, Tiziana Annese, Mariella Errede, Giorgina Specchia, Rebecca Senetta, Paola Cassoni, Domenico Ribatti, Beatrice Nico

**Affiliations:** ^1^ Department of Basic Medical Sciences, Neurosciences and Sensory Organs, Section of Human Anatomy and Histology, University of Bari Medical School, Bari, Italy; ^2^ Division of Medical Genetics, Department of Biomedical Sciences and Human Oncology (DIMO), University of Bari Medical School, Bari, Italy; ^3^ Department of Emergency and Transplantation, Section of Hematology, University of Bari Medical School, Bari, Italy; ^4^ Department of Biomedical Sciences and Human Oncology, University of Turin Medical School, Turin, Italy; ^5^ National Cancer Institute “Giovanni Paolo II”, Bari, Italy

**Keywords:** angiogenesis, cancer stem cells, endothelium, lymphoma, Stat3

## Abstract

With the aim of elucidating the relationship between Stat3 expression and tumor vessels abnormalities in the PCNLs, in this study we evaluated Stat3 and pStat3 expression by Real-time PCR and by immunohistochemistry in biopsy sections from PCNSL patients. Correlations of the expression levels with the presence of aberrant vessels were analyzed by confocal laser microscopy analysis, using FVIII as endothelial cell marker, CD133 and nestin as cancer stem cell (CSC) marker, CD20 as tumor cell marker, and Stat3. In addition, we investigated Stat3 mutations in lymphoma cells to clarify the role of the constitutive expression of Stat3 and of its phosphorylated forms. Results showed that in PCNSL, putative endothelial cells lining the vessels are heterogeneous, expressing FVIII/ pStat3/CD133 (presumably originally they are vascular progenitor cells), as well as FVIII/CD20/CD133 (presumably originally they are tumor cells). Finally, we detected a fraction of the FVIII^+^ endothelial cell that co-expressed Stat3 bearing a tetraploid karyotype, while no amplification signal for the Stat3 gene was detected.

## INTRODUCTION

Primary Central Nervous System Lymphoma (PCNSL) has an aggressive course and poor prognosis, with a median survival of less than 20–24 months [1, 2]. PCNSL accounts for 1–3% of all primary brain tumors [3], but its incidence has increased in recent decades. The majority of PCNSL are diffuse large B-cell Lymphomas [4] and lymphomatous cells are typically localized in the perivascular areas [3].

Angiogenesis has a prognostic value in human NHLs [5], and PCNSL [6]. Expression of vascular endothelial growth factor (VEGF) in PCNSL cells is correlated with microvascular density, with longer survival and blood-brain barrier alterations [6]. We have previously demonstrated that different type of cells are involved in the formation of vascular wall in PCNSL [7]. Cancer-like stem cells (CSCs) may be involved in formation of tumor vessels, including brain tumors, through a cross-talk between endothelial cells and CSCs [8–10].

The signal transducer and activator of transcription 3 (Stat3) is involved in angiogenesis [11]. Janus kinase (JAK) phosphorylates Stat proteins, and active Stat3 contributes to the malignant phenotype [12]. Activated Stat3 promotes tumor cell proliferation and survival, immune suppression, invasion, and angiogenesis [13]. A higher Stat3 activation in tumor cells is associated with lower survival rates of patients with several malignant tumors [14–17]. High levels of phosphorylated Stat3 (pStat3) protein were found in diffuse large B-cell lymphoma [18, 19], while data on the expression of Stat3 in PCNSL are controversial [20–22]. Moreover, Stat3 favors proliferation and maintenance of CSCs, and its inhibition blocks tumor formation in glioblastoma [23].

With the aim of elucidating the relationship between Stat3 expression and tumor vessels abnormalities in the PCNSL, we evaluated Stat3 and pStat3 expression by Real-time PCR and by immunohistochemistry in biopsy sections from PCNSL patients. Correlations of the expression levels with the presence of aberrant vessels were analyzed by confocal laser microscopy analysis, using FVIII as endothelial cell marker, CD133 and nestin as CSC marker, CD20 as tumor cell marker, and Stat3. In addition, we investigated Stat3 mutations in lymphoma cells to clarify the role of the constitutive expression of Stat3 and of its phosphorylated forms.

## RESULTS

### Endothelial and tumor cells express Stat3

**Figure 1:**
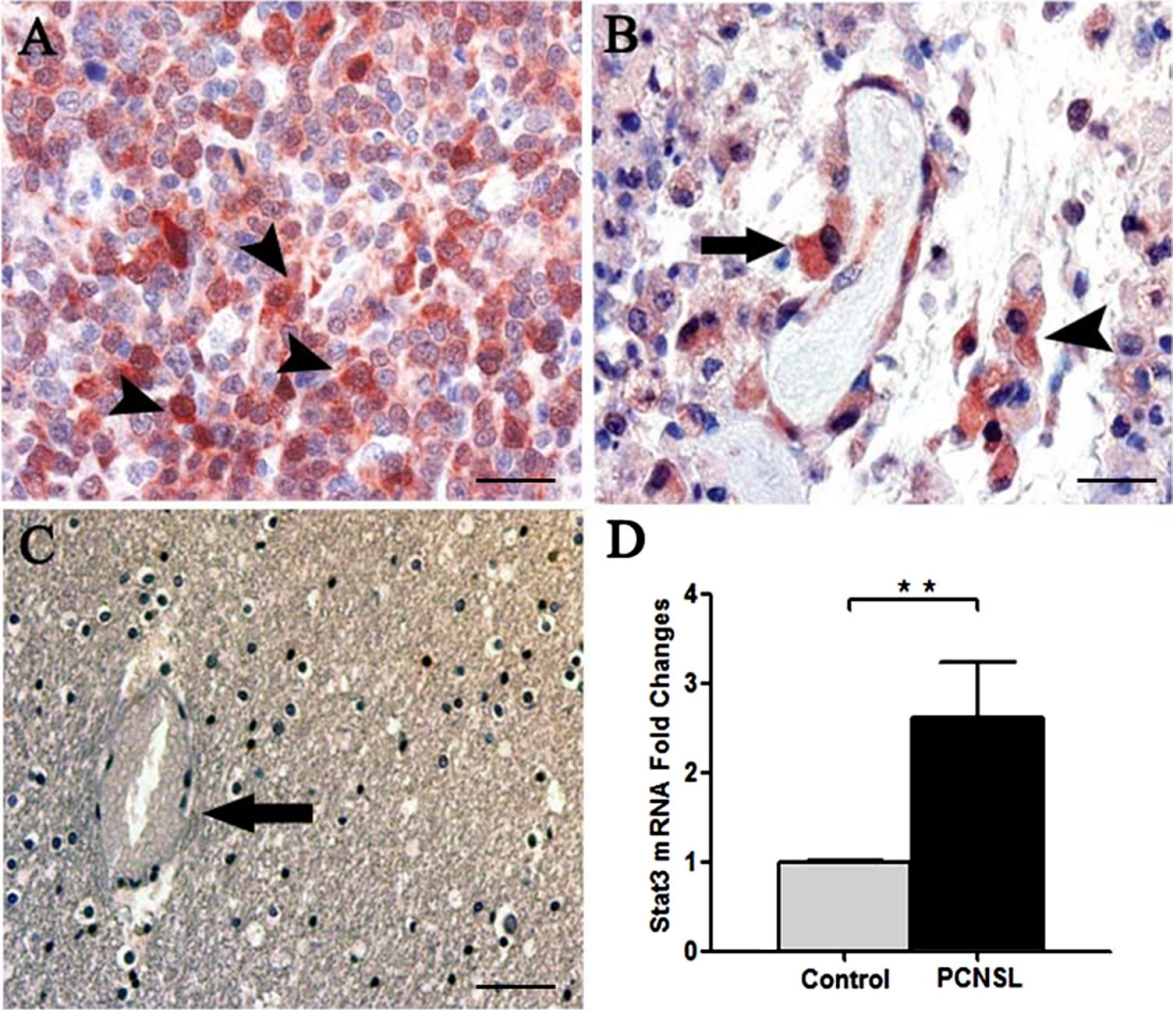
Stat3 Immunohistochemistry (**A**–**C**) and Stat3 messenger expression analysis (**D**). PCNSL section showing a strong Stat3 expression in the tumor cells (A, arrowhead) and in endothelial cells (B, arrow) lining a vessel near a cluster of Stat3 labeled tumor cells (B, arrowhead). Control brain section (C) showing a vessel (arrow) and Stat3 negative tumor cells. RT-PCR analysis (D) showing a significant Stat3 overexpression in PCNSL (2.623 ± SEM 0.305) compared to control brain (1.00 ± SEM 0.011) (**p < 0.001 vs Control). Scale bar: A–B 20 μm, C 40 μm.

**Figure 2:**
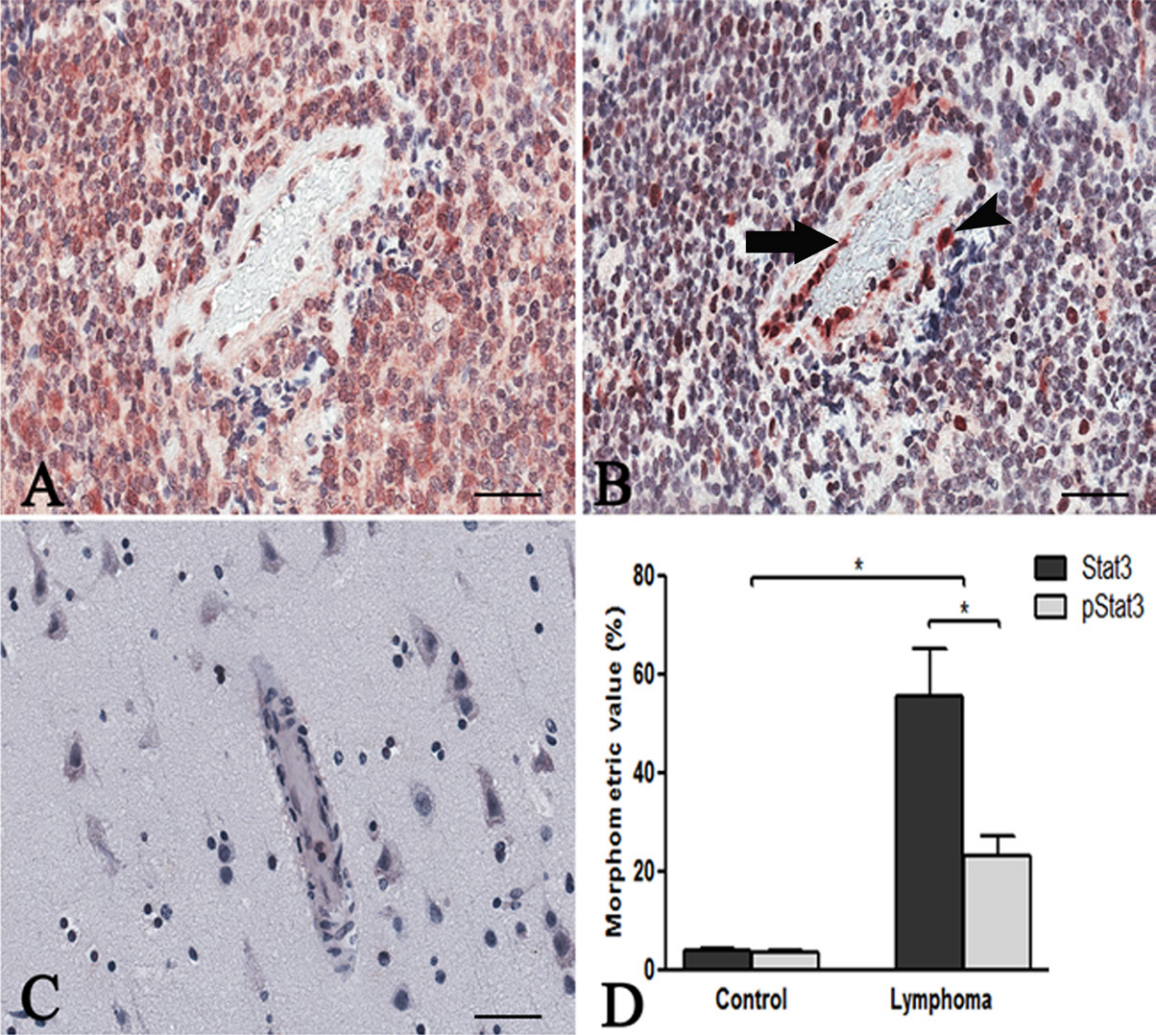
Stat3 and pStat3 immunohistochemistry PCNSL serial sections showing the same area (**A**, **B**) with a higher Stat3 cytoplasmic tumor expression (A) compared to pStat3 labeling of the endothelial cells lining a vessel (B, arrow) and of tumor perivascular cells (B, arrowhead). Control brain section showing negative pStat3 expression (**C**). Morphometric analysis (**D**) reveals a significant increase of Stat3 (55.51 ± SEM 9.414 ) and pStat3 (23.17 ± SEM 3.88) protein in PCNSL compared to control brain (3.7 ± SEM 0.52; 3.6 ± SEM 0.10) and a higher Stat3 expression compared to pStat3. (*p < 0.05) in PCNSL. Scale bar: A–C 40 μm.

**Figure 3:**
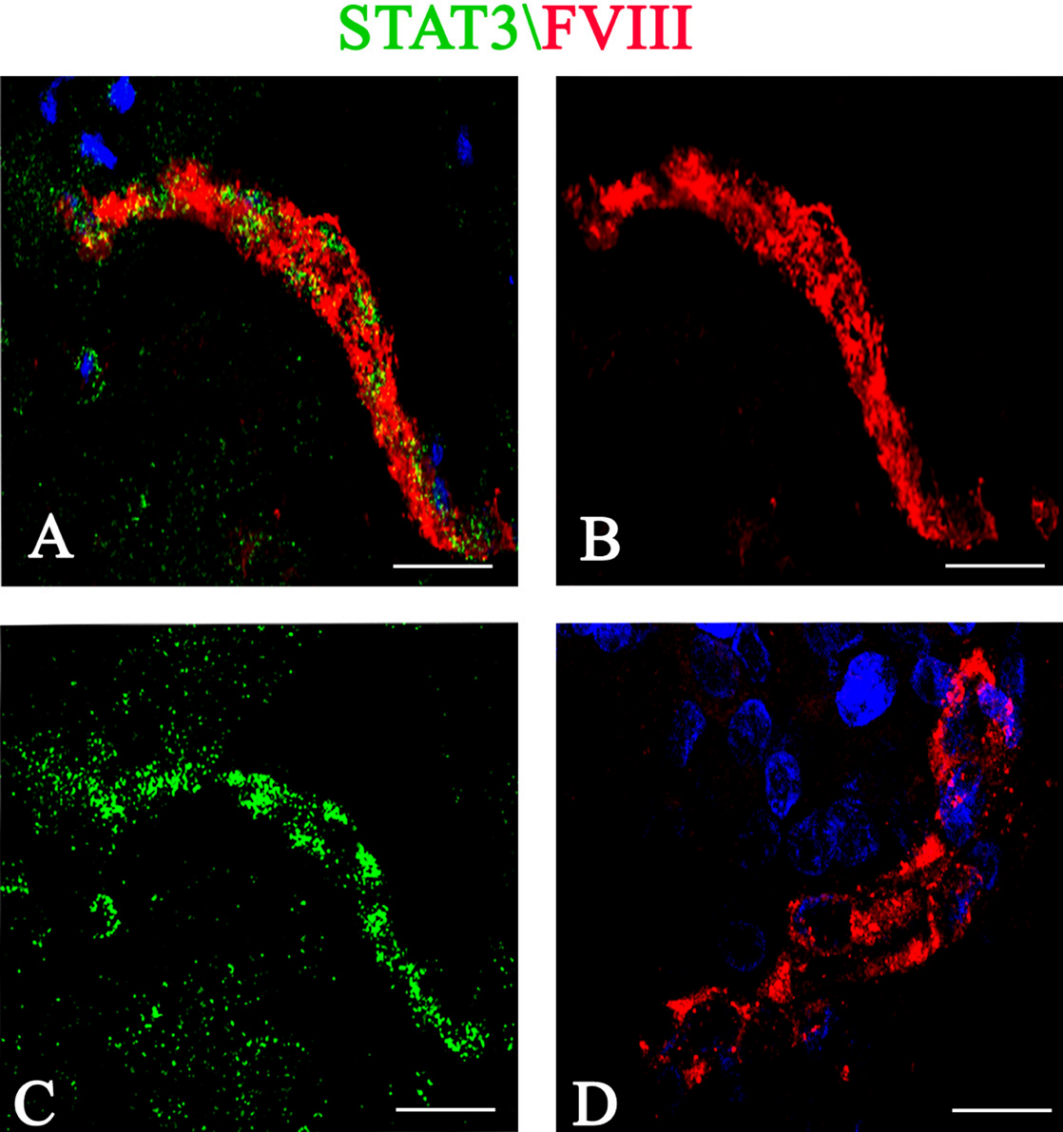
Stat3 (green)/FVIII (red) confocal dual immunofluorescence reaction Tumor vessels are lined by endothelial cells expressing FVIII and Stat3 fluorescence signals (**A**–**C**). Vessels from control brain section show only the FVIII signal (**D**). Scale bar: A–C 20 μm; D 12 μm.

Stat3 immunohistochemical expression showed high levels of total and phosphorylated Stat3 protein in tumor brain compared to normal brain. Stat3 showed an intense staining in tumor tissues (Figure 1A) compared to normal brain lacking Stat3 labeling (Figure 1C). Tumor vessels were lined by Stat3^+^ cells and tumor cells were identified near to the vessel wall (Figure 1B). After Real-time PCR (Figure 1D) a significant difference in Stat3 expression between tumor and control brain tissues was observed. To better identify changes in the expression of phosphorylated and total form of Stat3, immunohistochemical reactions were performed in the same areas of tumor tissue. Stat3 displayed a higher expression (Figure 2A) compared to pStat3, which strongly labeled the nuclei of the endothelial cells and clusters of tumor perivascular cells in the same area (Figure 2B). pStat3 was not detected in the control brain tissue (Figure 2C). Morphometric analysis showed an increased expression of Stat3 and pStat3 in the tumor brain tissues compared with control tissues (Figure 2D). After dual confocal immunofluorescence reactions, tumor vessels appeared lined by endothelial cells expressing both FVIII and Stat3 signals (Figure 3A–3C) while in the control brain tissue only FVIII^+^ vessels were detectable (Figure 3D).

### Endothelial tumor cells express CD133 and nestin stem cells marker

**Figure 4:**
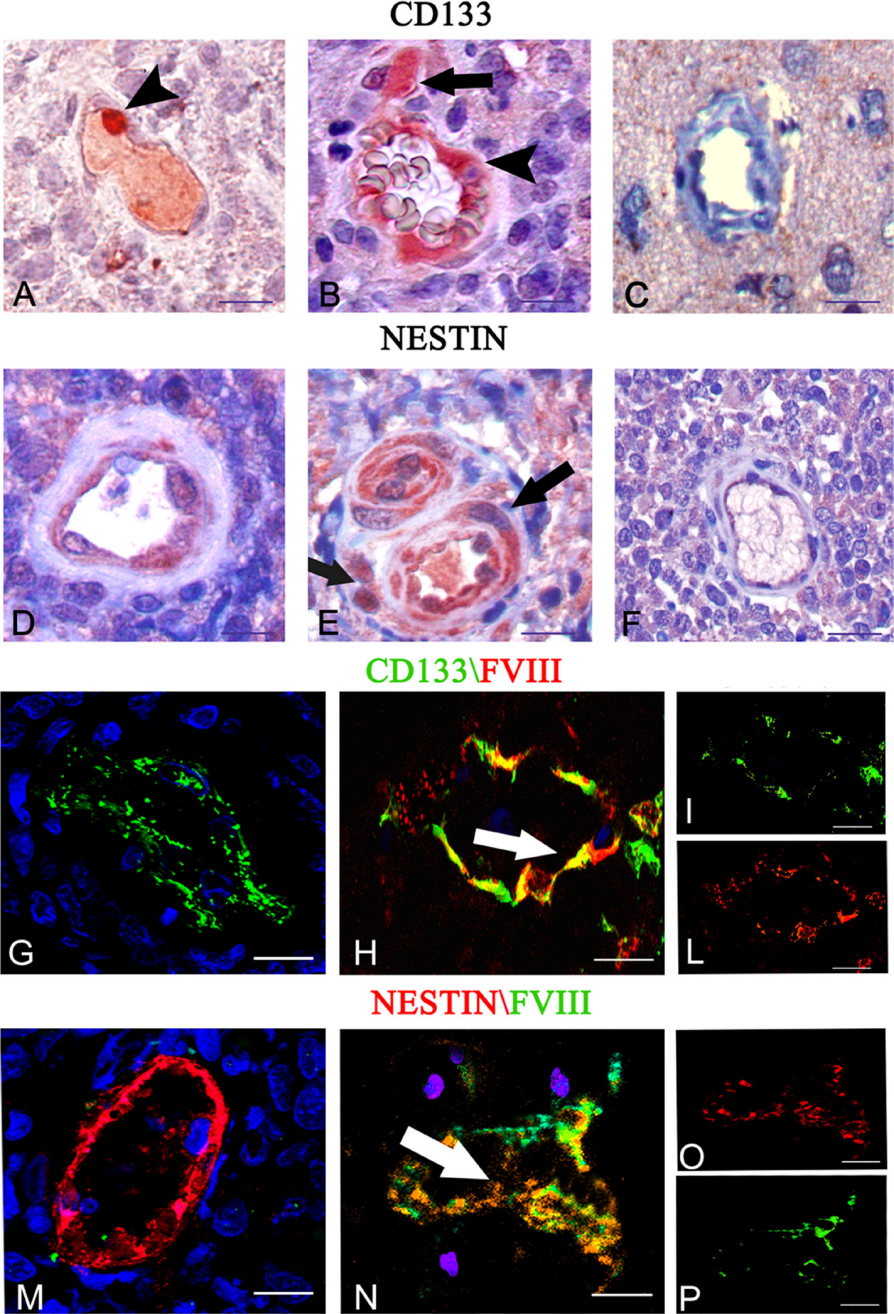
CD133 and nestin immunohistochemistry (**A**–**F**) and CD133 (green)/FVIII (red) (**G**–**L**), and nestin (red) /FVIII (green) (**M**–**O**) confocal dual immunofluorescence reaction. (A–E) Tumor vessels lined by CD133 (A–B arrowhead) and nestin (D–E) labeled endothelial cells near to CD133 (B, arrow) and nestin (E, arrow) labeled tumor cells. Control brain section showing unlabeled vessel and cells. (C, F). (G–P) Tumor endothelial cells co-expressing FVIII (H, L red) and CD133 (H, I green), and nestin (N, O red) and FVIII (N, P green) with orange colocalization signals (H, N arrows). FVIII^−^ tumor vessels express only CD133(G) and Nestin (M) markers. Scale bar: A–F, 10 μm; G–P 12,5 μm.

After immunohistochemistry, tumor vessels appeared lined by CD133^+^ (Figure 4A, 4B) and nestin^+^ (Figure 4D, 4E) cells and CD133^+^ and nestin^+^ tumor cells were detected near the vessels (Figure 4B, 4E). Instead, in the control brain tissue the vessels appeared both CD133^−^ and nestin^−^ (Figure 4C, 4F). After dual confocal immunofluorescence reactions, tumor vessels were lined by both CD133^+^ CSCs and FVIII^+^ endothelial cells (Figure 4H–4L), as well as nestin^+^ CSCs and FVIII^+^ (Figure 4N–4P) cells, with merged colocalization fluorescence signals. Moreover we identified CD133^+^/ FVIII^−^ vessels (Figure 4G) and nestin^+^/FVIII^−^ vessels (Figure 4M). The morphometric analysis showed that 14% ± 3 of vessels were CD133^+^/FVIII^−^and 86% ± 4 of vessels were CD133^+^/FVIII^+^, while 10% ± 2 of vessels were nestin^+^/FVIII^−^ and 90% ± 4 were nestin^+^/FVIII^+^.

### CD133-positive stem cells and CD20-positive tumor cells express pStat3 and endothelial FVIII marker

**Figure 5:**
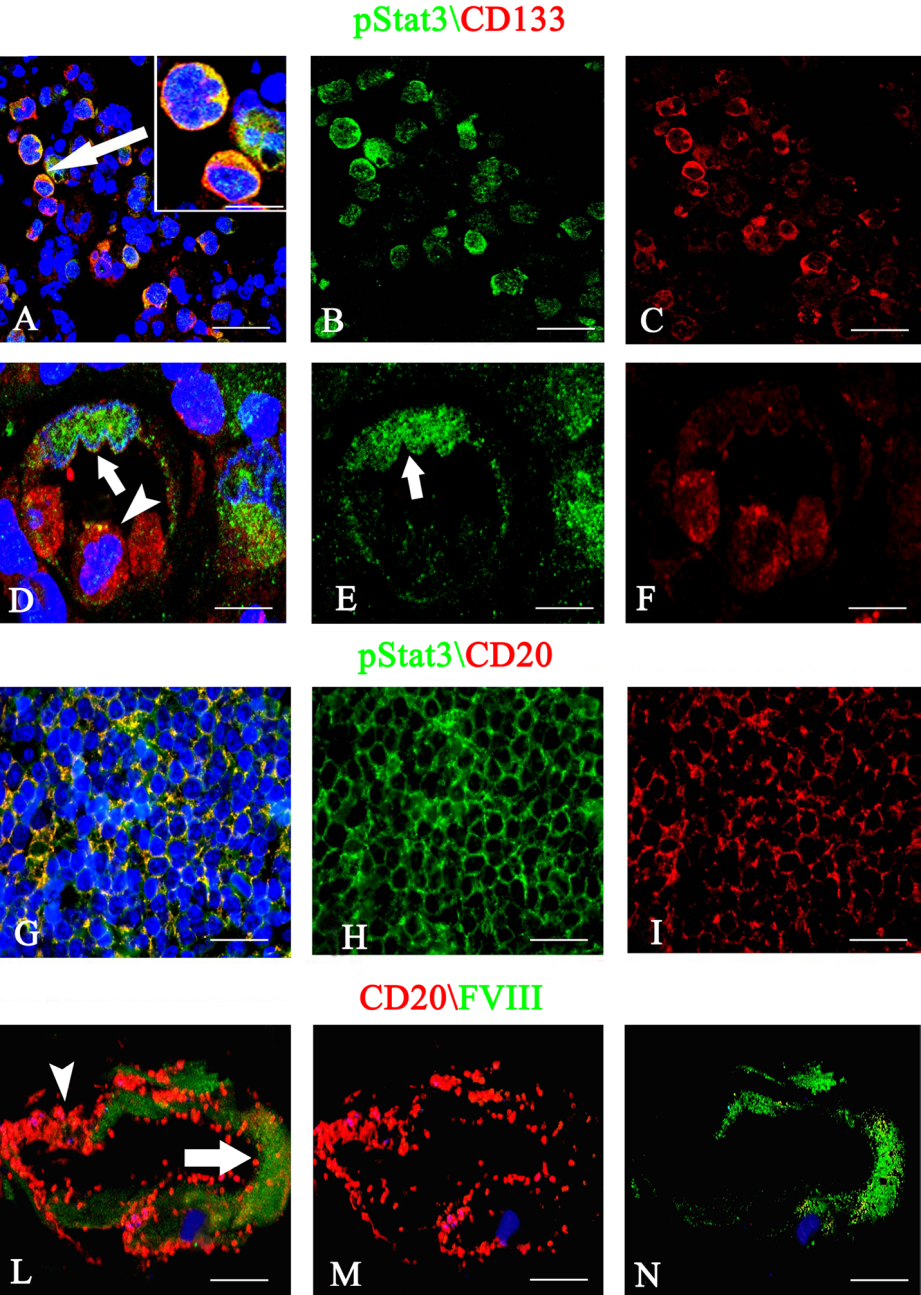
pStat3/CD133 (**A**–**F**), pStat3/CD20 (**G**–**I**) and CD20/FVIII (**L**–**N**) confocal dual immunofluorescence reaction. (A–C) Tumor cells labeled by anti-pStat3 (B) and anti-CD133 (C) antibodies with merged signal (A, arrow ). (D–F) Tumor vessel lined by green cells expressing pStat3 with both nuclear (D, E arrow) and cytoplasmic fluorescence and by red cells expressing CD133 (D, F) with some points of colocalization (D, arrowhead). (G–I) PCNSL sections showing CD20 (I) red tumor cells coexpressing pStat3 (G, H) and tumor vessel expressing green FVIII endothelial (L, arrow) and red CD20 tumor signals (L, arrowhead). Scale Bar: A–C 22 μm; A insert 15 μm, D–F 12 μm; G-I 20 μm; L–N 12 μm.

**Figure 6:**
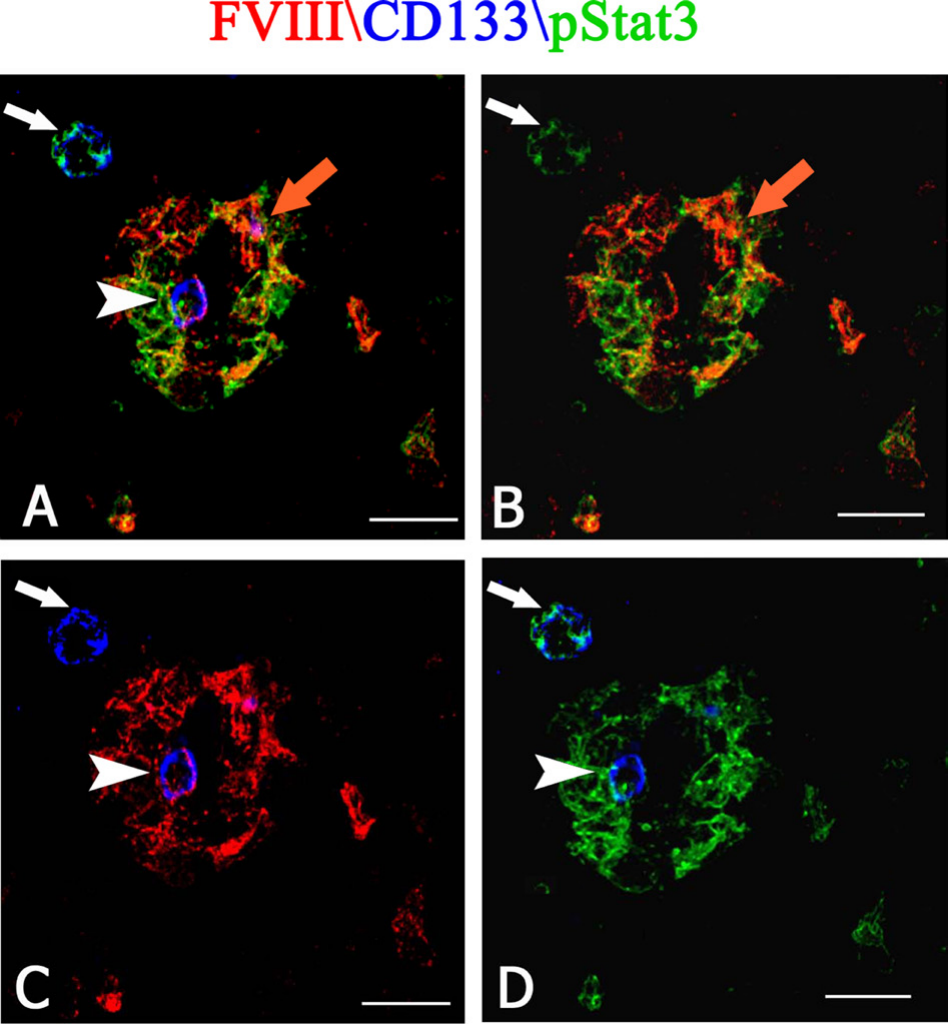
Multiple FVIII (red), CD133 (blue) and pStat3 (green) confocal immunofluoresce reaction PCNSL tumor vessels lined by FVIII positive endothelial cells (**A**, **B**, **C** red signal) and expressing both pStat3 ( A, B, **D** green signal) and CD133 ( A, C, D blue signal). An orange signal of FVIII and pStat3 colocalization (A, B orange arrow) is present. Note a CD133 blue cells (A, C, D, arrowhead) expressing FVIII red (A, C arrowhead) and pStat3 (D arrowhead) green fluorescence in the vessel and a CD133 blue cells (C, arrow) expressing pStat3 (A, B, D arrow) near the vessel. Scale bar: A–D 8 μm.

After dual confocal immunofluorescence reactions, pStat3 was expressed by CD133^+^ cells in the tumor tissue, showing an orange colocalization signal in the cytoplasm (Figure 5A–5C). The morphometric analysis showed that the pStat3 and CD133 labeled cells were significantly larger than unlabeled ones (11,8 ± 1,8 vs 6,2 μm diameter, *p* < 0.0001). Moreover, pStat3 and CD133 were colocalized in the tumor vessels, pStat3 showing both cytoplasmic and nuclear expression (Figure 5D, 5E). After confocal analysis, CD20^+^ tumor cells appeared also pStat3^+^ (Figure 5G–5I) and vessels labeled by FVIII^+^ and CD20^+^ cells were also detected (Figure 5L–5N). The multiple confocal immunofluorescence, confirmed the presence of tumor vessels lined by FVIII^+^ endothelial cells (Figure 6A–6C red signal) and expressing both pStat3 (Figure 6A, 6B, 6D green signal) and CD133 (Figure 6A, 6C, 6D blue signal).

**Figure 7:**
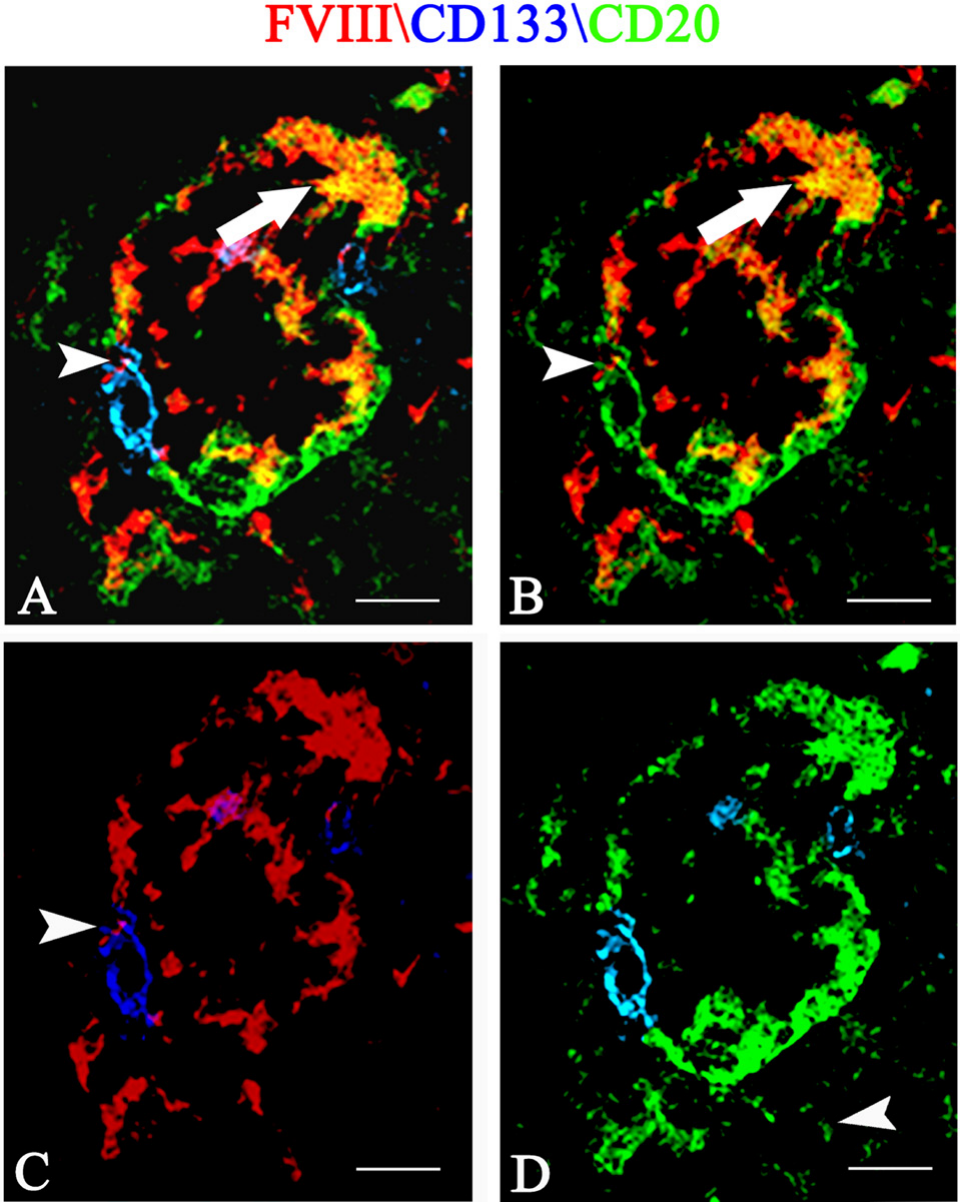
Multiple FVIII (red), CD133 (blue) and CD20 (green) confocal immunofluoresce reaction Tumoral vessel co-expressing FVIII endothelial (**A**, **B**, **C** red signal ), CD20 tumoral (A, B, **D**, green signal) and CD133 CSCs markers (A, C, D blue signal). A FVIII\CD20 orange colocalization is recognizable (A, B arrow) and a CD133 labeled cells (A, C, D blue signal) express both red endothelial (A, B, C arrowhead) and green CD20 tumoral (B, D green) fluorescence. Scale bar: A–D 12 μm.

Finally, after multiple immunofluorescence labeling, tumoral vessels co-expressing FVIII endothelial marker (Figure 7A, 7B, 7C red signal ), CD20 tumoral marker (Figure 7A, 7B, 7D, green signal) and CD133 CSCs marker (Figure 7A, 7C, 7D blue signal) were recognized.

**Figure 8:**
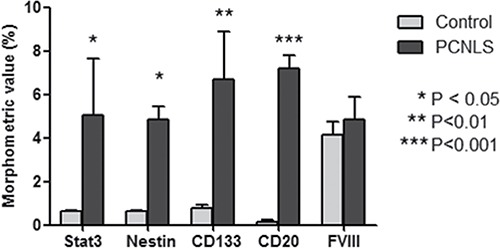
Morphometric analysis of Stat3, Nestin, CD133, CD20 and FVIII expression Morphometric analysis shows a significant increased expression of Stat3, CD133, Nestin and CD20 protein in PCNSL compared with control brain.

Overall, these data show, for the first time, that tumoral FVIII^+^ vessel in PCNSL are formed by CD20^+^/CD133^+^ cells (Figure 7) as well as that FVIII^+^ cells express both CD133 and pStat3 (Figure 6). Moreover, morphometric analysis shows a significant increased expression of Stat3, CD133, nestin and CD20 protein in PCNSL compared with control brain (Figure 8).

### Genetic analysis of Stat3 in PCNSL

**Figure 9:**
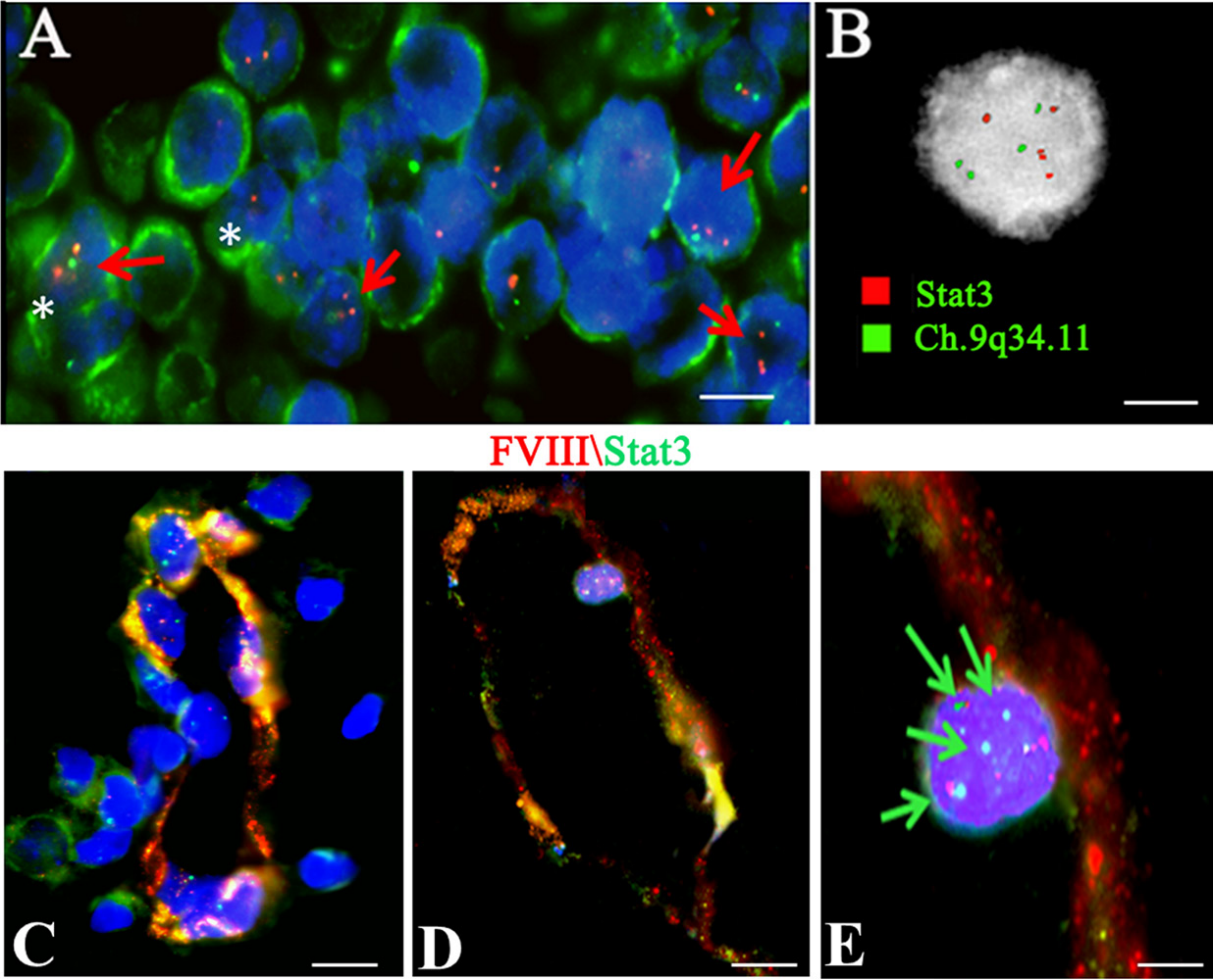
Tetraploid CD20 positive cells in PCNSL tissues and Tetraploid Stat3 positive cells in PCNSL vessels investigated by FICTION (**A**, **C**, **D**) and FISH (**B**) analysis. (A) Green CD20 tumor cells (A, white asterisk) show red and green aberrant fluorescent spots in the nuclei (A, red arrow). (B) Interphase Fish analysis showing four red (Ch: 17q21.31 Stat3) and four green (Ch: 9q34.11) fluorescent spots indicative of a tetraploid karyotype. (**C**–**E**) PCNSL vessel showing red FVIII and green Stat3 fluorescence with a tract showing an orange fluorescence signal. (E) FVIII/Stat3 labeled endothelial cells showing four green and red spot signals (green arrows). Scale bar: A 4 μm; B 2,5 μm; C–D 8 μm; E 3 μm.

To better understand the overexpression of Stat 3 in the vessel wall and tissues of PCNSL specimens, genetic analysis of the Stat3 gene was performed. Interphase FISH analysis and the FICTION technique on the FFPE tumor samples, and Stat3 gene sequencing on genomic DNA, were carried out. The FICTION technique detected red and green aberrant fluorescent spots in the nuclei of CD20^+^ cells (Figure 9A). In detail, the Interphase FISH analysis, showed four red (Chr:17q21.2, Stat3) and four green (Chr:9q34.11) fluorescent spots in the nuclei of PCNSL cells, indicative of a tetraploid karyotype (Figure 9B). After FICTION with FVIII and Stat3 dual immunofluorescence reaction, and FISH with the Stat3 gene (red) and chromosome 9q34.11 (green) probes, tumor vessels showed a merge fluorescent colocalization signal in some vascular tracts, while in other tracts the signals were separate (Figure 9C–9D). Moreover, Stat3^+^ cells with a tetraploid karyotype were detected in the vessel wall (Figure 9E).

All Stat3 exons and adjacent intronic regions were sequenced on genomic DNA isolated from biopsy samples derived from PCNSL specimens, but no pathogenic variants were found. Only two Single Nucleotide Polymorfisms (SNPs) were detected, the Stat3 rs2293152 G-allele (minor allele count/MAF = 0.346) and rs3830585 dupT-allele (minor allele count/MAF = 0.402).

## DISCUSSION

Avascular phase followed by a vascular phase characterizes growth in both solid and haematological tumors [25, 26]. Different mechanisms are involved in tumor angiogenesis: sprouting angiogenesis; non-sprouting angiogenesis (intussusceptive microvascular growth); postnatal vasculogenesis and vasculogenic mimicry.

Mounting evidence has shown that Stat3 is strongly linked to tumor angiogenesis and metastasis [27, 28] and is related to poor prognosis in different tumors [15, 29]. Anti-tumor effects of Stat3 knockdown has been demonstrated by means of small interfering RNA (siRNA), micro-RNA (mi-RNA), or small molecule inhibitors [30–33]. Inhibitory molecules, which down-regulate Stat3 phosphorylation and induce apoptosis in tumor cells have been developed [34–42]. Moreover, previous studies have shown that metformin, an anti-diabetic drug, could inhibit Stat3 phosphorylation and reduce risk of development in many type of cancer [43, 44]. Therefore Stat3 is a prognostic indicator and a therapeutic target. In this study we observed pStat3 expression in tumor PCNSL vessels and cells, moreover we found high levels of total and phosphorylated Stat3 protein in tumor brain compared to normal brain. Elevated levels of the phosphorylated protein have been reported in human glioblastoma multiforme (GBM) samples [45] and in GBM cell lines [46]. IL-6 and IL-10 activate Stat proteins [47]. IL-6 contributes to tumor metastasis via the JAK/Stat3 signaling pathway [48, 49], and IL-10 phosphorylates of Stat3 in PCNSL cells [50], while the role of IL-6 in PCNSL remains poorly defined.

We found that pStat3 was elevated in PCNSL brain samples compared to normal tissues. Overexpression of Stat3, as shown by Real-time PCR and immunohistochemical reactions, might mediate the cellular response to cytokines and growth factors [51, 52].

In this study we also demonstrated Stat3 expression in FVIII^+^ endothelial cells that lined PCNSL vessels and in FVIII^+^ cluster of cells in PCNSL brain samples while in control samples endothelial cells were FVIII^+^. Moreover in tumor tissues, cells co-expressing pStat3 and CD133, and vessels co-expressing CD133, pStat3, CD20 and FVIII markers, were detected. Therefore, putative endothelial cells lining these vessels are heterogeneous, expressing FVIII/pStat3/CD133 (presumably originally they are vascular progenitor cells), as well as FVIII/CD20/CD133 (presumably originally they are tumor cells), as already suggested for CSCs [53]. Interestingly, Stat3 is required for the maintenance of pluripotency in murine stem cells [54–8], and is necessary for self-renewal of murine neural stem cells [58, 59]. Neural stem/progenitor cells has been identified in bioptic specimens from different tumors [60]. Moreover, neural stem cells and progenitors cells have been labeled by nestin [61] and CD133 [62, 63].

The data are in agreement with previously published studies showing that tumor cells can give rise to tumor-associated endothelial microvessels in human B-cell lymphomas, multiple myeloma, and malignant melanoma [64–67]. In this work we have demonstrated, for the first time, that FVIII positive tumor vessels are formed also by CD133, nestin and pStat3 positive cells, suggesting that in PCNSL CSCs might contribute to the tumor vasculature. CSCs reside in a vascular niche named the CSC niche [68], and stimulate tumor angiogenesis. Tumor vasculature, in turn, supports CSC self-renewal and maintenance. CSCs produce high levels of VEGF [69], and recruit endothelial precursors for revascularization and tumor re-growth [70].

In addition, we further analyzed the tumor vasculature by combining FVIII and Stat3 dual immunofluorescence reactions and fluorescence *in situ* hybridization using the Stat3 locus specific probe (Chr:17q21.2) and the probe for chromosome 9q34.11. We detected a fraction of FVIII^+^ endothelial cell that co-expressed Stat3, bearing a tetraploid karyotype, while no amplification signal for Stat3 gene was detected. This polyploidy, a common feature of tumor cells, supports the notion that the tumor vascular endothelium in our PCNSL samples is of neoplastic origin. We also identified the same chromosomal aberration in CD20^+^ tumor cells. These findings confirm that in PCNSL, either the tumor cells can differentiate into endothelial cells.

Genomic analysis of tumors demonstrates significant genetic intra- and inter-tumor heterogeneity [71]. Colorectal cancer endothelial cells overexpress specific transcripts as a result of qualitative differences in gene profiling as compared with endothelial cells of the normal colorectal mucosa [72]. Further studies in glioma [73] and in invasive breast carcinoma [74] demonstrated a distinct gene expression pattern related to tumor endothelial cells, which cells acquire genotype alterations, leading to altered anti-angiogenic targets and resistance [75], and the contiguity of tumor cells and endothelial cells may be responsible for the genotype alterations [76].

Overall, these data suggest that in PCNSL, putative endothelial cells lining the vascular wall are heterogeneous, and are formed by two cellular populations, expressing FVIII/ pStat3/CD133 (presumably originally they are vascular progenitor cells), as well as FVIII/CD20/CD133 (presumably originally they are tumor cells).

## MATERIALS AND METHODS

### Patients and tissue samples

The clinical and anatomical features of the patients investigated in this study are reported in Table 1. All patients were affected by histologically proven primary human diffuse large B-cell PCNSL. Surgical specimens were fixed in 4% formaldehyde, routinely processed and paraffin-embedded. Three samples of histologically normal brain, removed in the course of surgical exposure, were used as control. The study was approved by the local Ethics Committee at the University of Bari Medical School, and all the patients gave their informed consent in accordance with the declaration of Helsinki.

**Table 1:** Clinical and anatomical features of 20 PCNSL patients^†^

Case	Sex	Age (years)	Tumor location	Ki 67*
1	M	40	Parieto-frontal lobe	80
2	M	74	Occipital lobe	75
3	M	82	Occipital lobe	40
4	M	69	Parietal lobe	95
5	M	72	Parieto-temporal lobe	60
6	M	58	Temporal lobe	70
7	M	72	Basal ganglia	90
8	F	65	Cerebellar	80
9	M	72	Frontal lobe and corpus callosum	75
10	F	75	Temporal lobe	70
11	M	66	Frontal lobe	85
12	M	64	Parieto-frontal lobe	80
13	M	73	Parieto-frontal lobe	80
14	F	38	Multiple intra-cerebral	65
15	M	58	Occipital lobe	80
16	M	72	Parietal lobe	45
17	F	64	Parieto-occipital lobe	80
18	M	37	Basal ganglia	80
19	F	67	Basal ganglia	80
20	M	44	Cerebellar	40

*Ki -67 tumor proliferative index.

^†^All the patients have high degree of tumor and have been treated with a classic schema of chemotherapy, including methotrexate + Cytarabine (araC).

### Stat3, pStat3, CD133 and nestin immunohistochemistry

Four μm thick histological sections collected on poly-L-lysine-coated slides (Sigma-Aldrich, St. Louis, MO, USA), were deparaffinized and stained with an automated immunostainer (Autostainer, Dako, Glostrup, Denmark) with a dextran polymer-based system method (EnVisionTM Flex^+^, Dako) using 3′-3′diaminobenzidine as chromogen. The sections were rehydrated in a xylene-graded alcohol scale and then rinsed for 10 minutes in 0.1M PBS. For Stat3, pStat3, CD133 and nestin immunodetection, sections were pretreated with sodium citrate pH 6.1 (Dako) solution for 30 minutes at 98°C in Dako PT Link for antigen retrieval, and then incubated with goat polyclonal anti-Stat3 (ab5073, Abcam discover more, Cambridge, UK), mouse monoclonal anti-pStat3 (sc56747, Santa Cruz Biotechnology, Santa Cruz, CA, USA), rabbit polyclonal anti-CD133 (ab19898, Abcam) and mouse monoclonal anti-nestin (sc23927, Santa Cruz Biotechnology), diluted 1:50, 1:100, 1:200, 1:500 respectively. Thereafter, the sections were counterstained with Mayer hematoxylin and mounted in synthetic medium. Specific preimmune serum (Dako), replacing the primary antibodies, served as negative control.

### Morphometric analysis of Stat3 and pStat3 expression

For each case, three slides stained for Stat3 and pStat3 expression were scanned using the whole-slide scanning platform Aperio Scanscope CS (Leica Biosystems, Nussloch, Germany). All the slides were scanned at the maximum available magnification (40 ×) and stored as digital high resolution images on the workstation associated with the instrument. Digital slides were inspected with Aperio ImageScope v.11 software (Leica Biosystems, Nussloch, Germany) at 20 × magnification and ten fields with an equal area were selected for the analysis at 40 × magnification. Stat3 and pStat3 expression was assessed with the Positive Pixel Count algorithm embedded in the Aperio ImageScope software and reported as positivity percentage, defined as the number of positively stained pixels on the total pixels in the image. The statistical significance of differences between the mean values of the percent labeled areas between patients and control brain tissues was determined by the 2way Anova test in GraphPad Prism 5.0 software (GraphPad software, La Jolla, CA, USA). Findings were considered significant at *P* values < 0.05.

### Real time PCR

Total RNA was extracted from FFPE blocks tissues of 20 patients and 3 normal control brain, using RecoverAll™ Total Nucleic Acid isolation kit (Ambion, Life Technologies, Inc., Austin, TX,USA) and then used to synthesize the first-strand c-DNA with the IScriptcDNA Synthesis kit (Bio-Rad Laboratories, Hercules, CA, USA), according to the manufacturer's instructions. For the detection of Stat3 expression, cDNA was amplified with the iTaq SYBR Green supermix using a ROX kit (Bio-Rad Laboratories). PCR amplification was performed using the Chromo4 real-time PCR Detection System (Bio-Rad Laboratories). Samples were normalized to human RPLPO (large ribosomal protein PO). Table 2 show the sequences of primers (Sigma-Aldrich) used for Stat3 amplification.

**Table 2:** Primer sequences for real-time PCR

Stat3/S	5′ GCTGGCTGACTGGAAGAG3′
Stat3/AS	5′ AGTTGAGATTCTGCTAATGACG3′
RPLPO/S	5′ CCTTCCCACTTGCTGAAAAGG 3′
RPLPO/AS	5′ ACAAAGGCAGATGGATCAGCC 3′

### Dual and triple immunofluorescence-confocal laser scanning microscopy

Twelve micrometer thick deparaffinized brain sections were incubated for 30 minutes in a blocking buffer [BB; phosphate-buffered saline (PBS), pH 7.4, 1% bovine serum albumin, 2% fetal calf serum] and exposed to primary antibodies: (i) goat anti-Stat3 (ab5073, Abcam) and rabbit anti-FVIII (A0082, Dako), diluted 1:100 and 1:50 in BB, respectively, overnight at 4°C; (ii) mouse anti-pStat3 (sc56747, Santa Cruz Biotechnology) and rabbit anti-CD133 (ab19898, Abcam), diluted 1:50 and 1:5 in BB, respectively, overnight at 4°C; (iii) mouse anti-CD133 (130-090-422, MACS Miltenyi Biotec, Bergisch Gladbach, Germany) and rabbit anti-FVIII (A0082, Dako), diluted 1:5 and 1:50 in BB, respectively, overnight at 4°C; (iv) rabbit anti-pStat3 (ab30647, Abcam) and mouse anti-CD20 (M0755, Dako), diluted 1:50 and 1:200 in BB, respectively, overnight at 4°C; (v) mouse anti-CD20 (M0755, Dako) and rabbit anti-FVIII (A0082, Dako), diluted 1:200 and 1:50 in BB, respectively, overnight at 4°C; (vi) mouse anti-nestin (sc23927, Santa Cruz Biotechnology) and rabbit anti-FVIII (A0082, Dako) diluted 1:50 in BB overnight at 4°C; (vii) rabbit anti-FVIII (A0082, Dako), mouse anti-CD133 (130-090-422, MACS Miltenyi Biotec) and mouse anti-pStat3 (sc56747, Santa Cruz Biotechnology), diluted 1:50, 1:100 and 1:100 in BB, respectively, overnight at 4°C; (viii) rabbit anti-FVIII (A0082, Dako Corporation), mouse anti-CD133 (130-090-422, MACS Miltenyi Biotec) and mouse anti-CD20 (M0755, Dako), diluted 1:50, 1:100 and 1:200 in BB, respectively, overnight at 4°C After washing in PBS the sections were incubated for 2 hours with the secondary antibodies: (i) Alexa Fluor 488 donkey anti-goat and Alexa Fluor 555 donkey anti-rabbit antibodies (Invitrogen, Carlsbad, CA, USA), diluted 1:300 in BB for Stat3/FVIII dual localization; (ii) Alexa Fluor 488 goat anti-mouse and Alexa Fluor 555 goat anti-rabbit antibodies (Invitrogen) diluted 1:300 in BB for pStat3/CD133; (iii) Alexa Fluor 488 goat anti-mouse and Alexa Fluor 555 goat anti-rabbit antibodies (Invitrogen) diluted 1:300 in BB for CD133/FVIII; (iv) Alexa Fluor 488 goat anti-rabbit and Alexa Fluor 555 goat anti-mouse antibodies (Invitrogen), diluted 1:300 in BB for pStat3/CD20 dual localization; (v) Alexa Fluor 555 goat anti-mouse and Alexa Fluor 488 goat anti-rabbit antibodies (Invitrogen), diluted 1:300 in BB for CD20/FVIII dual localization; (vi) Alexa Fluor 555 goat anti-mouse and Alexa Fluor 488 goat anti-rabbit antibodies (Invitrogen), diluted 1:300 in BB for nestin/FVIII dual localization; (vii) Alexa Fluor 555 goat anti-rabbit, Alexa Fluor 488 goat anti-mouse and Alexa Fluor 647 donkey anti-mouse antibodies (Invitrogen), diluted 1:300 in BB for FVIII/CD133/pStat3 triple localization; (viii) Alexa Fluor 555 goat anti-rabbit, Alexa Fluor 488 goat anti-mouse and Alexa Fluor 647 donkey anti-mouse antibodies (Invitrogen), diluted 1:300 in BB for FVIII/CD133/CD20 triple localization. All the samples were incubated for 20 minutes with 0.01% TO-PRO-3 (Invitrogen) for nuclear staining and mounted in Vectashield (Vector Laboratories Inc., Burlingame, CA, USA). Negative controls, obtained by substituting primary antibodies with specific preimmune serum (Dako), showed no staining of the sections. The sections were examined under a Leica TCS SP5 (Leica,Wetzlar, Germany) confocal laser scanning microscope using 40 × and 63 × objective lenses with either 1 × or 2 × zoom factors. A sequential scan procedure was applied during image acquisition of the two fluorophores. Confocal images were taken at 200 nm intervals through the z-axis of the section covering a total depth of 10 μm. Images from individual optical planes and multiple serial optical sections were analyzed, digitally recorded and stored as TIFF files using Adobe Photoshop software (Adobe Systems Inc., San Jose, CA, USA). Morphometric analysis was performed by two independent observers on ten randomly selected fields observed at 63 × magnification by using Cell^F as image analysis software (Olympus Italia,Rozzano,Italy).

### Morphometric analysis of nestin^+^/FVIII^−^, nestin^+^/FVIII^+^, CD133^+^/FVIII^−^ and CD133^+^/FVIII^+^ vessels in PCNSL sections

nestin^+^/FVIII**^−^**, nestin^+^/FVIII^+^, CD133^+^/FVIII**^−^** and CD133^+^/FVIII^+^ vessels were evaluated from five optical fields randomly chosen for each PCNSL samples at 200 × magnification. The images were acquired using the confocal fluorescence microscope (Leica) with an integrated camera. The software Cell^F as image analysis (Olympus Italia,Rozzano,Italy) was applied for the vessel counting. The values were presented as percentage of positive vessels for each staining over the total vessels number. The data were expressed as mean value ± SD.

### Morphometric analysis of pStat3 and CD133 cell expression

The diameter of the pStat3\CD133 labeled and unlabeled cells were evaluated using Leica Confocal Multicolor Package (Leica Microsystems) on single optical planes of randomly chosen fields from ten sections per tumor and control samples with 63 × oil lenses. The measure was made by optimized contrast and brightness enhancement functions and digital filters. The Graph Pad Prim 5.0 statistical package (GraphPad Software, San Diego, CA, USA) was used for the analysis and *P* < 0.05 was considered as the limit for statistical significance. The data were expressed as mean value ± SD.

### FISH analysis

FISH evaluation of paraffin sections was carried out with specific BAC clones. In detail, the Stat3 gene identification was performed using a pool of two BAC clones RP11-915H22 (17q21.2, chr17:42,310,278-42,457,908) and RP11-1151C17 (17q21.2, chr17:42,166,634-42,327,743) (UCSC, https://genome-euro.ucsc.edu/; Dec. 2013 release); as genome ploidy control, a contig of three BACs mapping on 9q34.11 was used (RP11-5J2, chr9:129,650,477-129,813,833; RP11-409K20, chr9:129798948-129968911; RP11-138E2, chr9:129917913-130096675). PCNSL sample preparations were hybridized *in situ* with probes labeled directly with Cy3 and fluorescein by nick translation [24]. Briefly, 1000ng of each labeled probe was used for FISH cohybridization experiments; hybridization was performed at 37°C in 2 X standard saline citrate, 50% (vol/vol) formamide, 10% (wt/vol) dextran sulfate, 5mg COT1 DNA (Bethesda Research Laboratories, Gaithersburg, MD, USA), and 3 mg sonicated salmon sperm DNA in a volume of 10 ml. Post-hybridization washing was at 60°C (0.1 X standard saline citrate). Interphase nuclei were identified by DAPI (4′,6-diamidino-2-phenylindole staining). Digital images were obtained using a Leica DMRXA epifluorescence microscope equipped with a cooled CCD camera (Princeton Instruments, Boston, MA, USA). Cy3 (red; New England Nuclear, Boston, MA, USA), fluorescein (green; Fermentas Life Sciences, Milan, IT), and DAPI (blue) fluorescence signals, which were detected by using specific filters, were recorded separately as grayscale images. Pseudocoloring and merging of images were performed with Adobe Photoshop software (Adobe Systems Inc)

### Immunofluorescence and fluorescence *in situ* hybridization analysis (FICTION)

Fluorescence immunophenotyping and interphase cytogenetics (FICTION), a technique combining immunofluorescence and FISH, was carried out on 4-μm-thick paraffin sections of the PCNSL samples. For the identification of the chromosomal Stat3 signal in tumoral cells FISH assay was performed and then the sections were stained with either anti-CD20 antibody, a cytoplasmic tumoral cells marker, or with anti- FVIII, an endothelial cells marker, and anti-Stat3. In brief, deparaffinized and rehydrated slides were next incubated at 96°C in Tris/EDTA acid buffer solution for 15 minutes, washed in sterile water and treated in 0.01 N HCL solution at 37°C for 2 minutes. Enzymatic digestion was then performed by adding 200 μl 0.4% pepsin (Sigma-Aldrich) solution and incubating at 37°C for 7 minutes. Thereafter, tissue samples were washed with sterile water, dehydrated in ethanol, and air dried. Hybridization was done with the Stat3 probe and the centromeric probe of chromosome 9 as control. Probe and target were co-denatured at 75°C for 5 minutes, followed by overnight hybridization at 37°C on StatSpinThermoBrite (Abbott Molecular, Abbott Park, Illinois, USA). Post-hybridization washing was carried out at 72 ± 1°C in 0.4XSSC for 2 minutes and in 2XSSC/0.1% Tween at room temperature for 1 minute. Then the slides were counterstained and mounted with DAPI-Antifade (Cytocell, Tarrytown NY). After post-hybridization washing, immunostaining was performed. The tissues were treated with normal goat serum at room temperature for 30 minutes and incubated overnight with mouse monoclonal anti-CD20, rabbit polyclonal anti-FVIII (M0755 and A0082, Dako) and goat polyclonal anti-Stat3 (ab5073,Abcam) primary antibodies at 22°C then washed in PBS buffer and incubated at room temperature for 2 hours, with Alexa Fluor 488 goat anti-mouse, Alexa Fluor 555 donkey anti-rabbit and Alexa Fluor 488

donkey anti-goat (Invitrogen) antibodies, respectively, used as secondary reagent. Afterwards, the slides were washed, counterstained with DAPI (Invitrogen), and mounted in Vectashield (Vector Laboratories Inc.). Images were captured using an Olympus BX51 microscope fitted with an Olympus DP70 camera, equipped with filter sets for DAPI (nuclei counterstaining), FITC (cytoplasmic immunofluorescence signal and green nuclear FISH signal) and TRITC (cytoplasmic immunofluorescence signal and red nuclear FISH signal). Hybridization signals were counted in 200 morphologically intact nuclei for each sample.

### DNA extraction and sanger sequencing

Genomic DNA was extracted from biopsy sections derived from PCNSL specimens using the QIAamp Mini Kit (Qiagen, Hilden, Germany), according to the manufacturer's instructions, and quantified on a BioSpectrometer Plus (Eppendorf, Hamburg, Germany). The entire coding regions of Stat3 (RefSeq NM_139276.2), including all splice junctions and adjacent intronic sequences, were amplified by standard PCR protocols using Taq DNA Polymerase (Thermo Scientific, USA) and the primer pairs listed in Table 3. Direct sequencing was performed using the BigDye Terminator v1.1 Cycle Sequencing Kit (Applied Biosystems) according to the manufacturer's instructions on an ABI 310 Genetic Analyzer (Applied Biosystems). The sequence analysis software Alamut® (Interactive Biosoftware) was used to interpret variants. Online databases including dbSNP (Database the Single Nucleotide Polymorphism Database), 1000 Genomes, ClinVar, EXAC (Exome Aggregation Consortium), COSMIC (Catalogue of Somatic Mutations in Cancer), ESP (Exome Sequencing Project), as well as on line search engines (e.g. PubMed, LOVD), were used to search for previously described variants.

**Table 3:** Primers for the PCR amplification of *Stat3* (NM_139276.2)

GENE	EXON	Primer sequence
Stat3		
	2	F 5′ GGACTTGTGGTGAACATATGC 3′
		R 5′ CCTAACAATTTGGAGAGTCAC 3′
	3	F 5′ GTATGCGTCGGCTTCAGAGC 3′
		R 5′ AACACTAACACCCGACTCTGC 3′
	4	F 5′ ACATCTCCATGGTCTGCTGC 3′
		R 5′ CATTCCTCCCAGACCAGGG 3′
	5	F 5′ TAGTTCTGTGTTCACATGTGC 3′
		R TTAATGAAAGCTCCCTGCCC 3′
	6	F 5′ GGCTTCATTTGAATTCTCCTC 3′
		R 5′ AACACAAACTCACTTTCTAGAG 3′
	7	F 5′ AGTATTCCCTCAGGTCAAGG 3′
		R 5′ TGAAATCCCGCAAGTGAGCG 3′
	8	F 5′ TGGTTAGAGACAGTCTGAGG 3′
		R 5′ TCAGAATTCAATCTAGCTTTCG 3′
	9	F 5′ CCATCTCACCTGTATACATTC 3′
		R 5′ CTACCACGTGAGTCTTTAGG 3′
	10	F 5′ ACTTCTGGTCATGGCCGTG 3′
		R 5′ CCTCAGTAAAATCTCTACTGG 3′
	11	F 5′ CTTGGCCTATTTACCTGTTGG 3′
		R 5′ AATGATGTCTGTCAAAGTTCTC 3′
	12	F 5′ CAGTAAATAACAGGTGGTCAAAG 3′
		R 5′ CAATCAACTATGTAGGTGACC 3′
	13	F 5′ GCTACTTGGTCACCTACATAG 3′
		R 5′ AAAGGCAGGTGTCCTGTGAG 3′
	14	F 5′ ATTCAAACACTTGGTATGTGGG 3′
		R 5′ GGTTGATGTTTCTAATTCTGGG 3′
	15	F 5′ TTACAGGCATGAGCCACCAC 3′
		R 5′ GAAGTTTTTGTCCTGAGTCACC 3′
	16	F 5′GATTTCCAAGGCTGTGAGAGC 3′
		R 5′ ACCCCTAAGTCGCAAGAGATC 3′
	17	F 5′ GTACCTAGTATAGACAATGAGC 3′
		R 5′ CTTTTCTGGGCGGGTGGG 3′
	18	F 5′ AATAACCTCTTGACCCCAAGC 3′
		R 5′ AGCCGTGCAGGTGAGCATTC 3′
	19	F 5′ GCAGTAGACTTGGCTTTCCC 3′
		R 5′ GGGACTTGGTTACATCTGTGC 3′
	20	F 5′ TGCCCTGTTAGCAATAACAAC 3′
		R 5′ CAACTAGAAGCAGTGATGAGG 3′
	21	F 5′ TGAGATGACCTAGCTGTAGG 3′
		R 5′ TCCAAGGATCCCAAAATTTCC 3′
	22	F 5′ CACAGTCAGTAAGAAAACTGG 3′
		R 5′ ACCTTTGGCAGATTAACTCTC 3′
	23	F 5′TTCCATTGTGTCTTGTCAACC 3′
		R 5′ CTCGGTGTGTACATGTGAGAG 3′
	24	F 5′ CAGAGGGTGGACAATGAAC 3′
		R 5′ TAGTAGTTTCAGATGATCTGGG 3′

### Statistical analysis

Data are reported as means ± SEM. Student's t-test was used for two-group comparisons and Newman-Keuls multiple comparison post-test was used to compare all treatment groups following one-way ANOVA. The Graph Pad Prim 5.0 statistical package (GraphPad Software, San Diego, CA, USA) was used for the analysis and *P* < 0.05 values were considered statistically significant.
